# Reactive Oxygen Species (ROS) in Metabolic Disease—Don’t Shoot the Metabolic Messenger

**DOI:** 10.3390/ijms26062622

**Published:** 2025-03-14

**Authors:** Ross T. Lindsay, Christopher J. Rhodes

**Affiliations:** 1 Research and Early Development, Cardiovascular, Renal and Metabolism, BioPharmaceuticals R&D, AstraZeneca, Gaithersburg, MD 20878, USA; 2 Research and Early Development, Cardiovascular, Renal and Metabolism, BioPharmaceuticals R&D, AstraZeneca, Cambridge CB2 0AA, UK

**Keywords:** reactive oxygen species (ROS) signalling, cellular second messenger, peroxisome, mitochondria, endoplasmic reticulum (ER), oxidation, metabolic disease

## Abstract

Reactive oxygen species (ROS) are widely considered key to pathogenesis in chronic metabolic disease. Consequently, much attention is rightly focused on minimising oxidative damage. However, for ROS production to be most effectively modulated, it is crucial to first appreciate that ROS do not solely function as pathological mediators. There are >90 gene products specifically evolved to generate, handle, and tightly buffer the cellular concentration of ROS. Therefore, it is likely that ROS plays a role as integral homeostatic signalling components and only become toxic in extremis. This review explores these commonly overlooked normal physiological functions, including how ROS are generated in response to environmental or hormonal stimuli, the mechanisms by which the signals are propagated and regulated, and how the cell effectively brings the signal to an end after an appropriate duration. In the course of this, several specific and better-characterised signalling mechanisms that rely upon ROS are explored, and the threshold at which ROS cross from beneficial signalling molecules to pathology mediators is discussed.

## 1. Introduction

Chronic “oxidative stress” is commonly considered a major contributor to the pathogenesis of metabolic diseases, such as obesity-linked type 2 diabetes, metabolic dysfunction-associated steatohepatitis (MASH), and associated complications like diabetic kidney and heart disease. The concept is that overly nutritious states lead to metabolic dysfunction, resulting in uncontrolled production of Reactive Oxygen Species (ROS). These ROS are then proposed to indiscriminately oxidise proteins, lipids, and other cell components, presaging cellular dysfunction, chronic inflammation, and fibrosis [1,2]. Extreme case scenarios, such as cardiac ischaemia, have justifiably led many investigators to note a correlation between ROS production levels and the severity of end-stage pathologies. Yet responding to these observations by seeking to minimise ROS production wherever it is found may fatally overlook the many valuable cellular functions ROS perform. Multiple disappointing, and mostly ineffective, clinical trials of antioxidant-based therapies to date [3,4,5,6] support the premise that abolition of all ROS would be every inch as detrimental as many believe its presence to be.

Despite ROS being widely maligned as bad actors on the cellular stage, much evidence indicates that they also perform many functions that are integral to cellular health and homeostasis [7,8,9]. At least 90 different genes encode cellular enzymes with the purpose of efficiently handling ROS (Supplementary Table S1), and highly conserved motifs feature prominently on other proteins that respond to their presence. It seems, therefore, that natural selection has favoured the generation of a complex ROS signalling system above eliminating ROS from metabolism, which would have been the likely outcome if they were solely toxic. With this in mind, perhaps it is time to better consider reactive oxygen species signalling (ROS-S) as a necessary and effective secondary messaging pathway, every bit on a par with established second messengers such as Ca^2+^ and cAMP. In this latter regard, it should also be noted that, as with ROS, if there are uncontrolled increases in [Ca^2+^]_i_ or [cAMP]_i_, then this too can be pathogenic [10,11,12,13]. Here, as an additional perspective to overviews associating excessive ROS production with “oxidative stress” as previously put forward [1,2], we concentrate on the necessity for ROS-S systems, with a focus upon some of their crucial roles in normal endocrine cardio-metabolic physiology.

## 2. The Biochemistry and Detection of ROS

The title Reactive Oxygen Species collectively characterises those molecules containing oxygen that are more reactive than oxygen itself [14]. ROS often contain a highly reactive unpaired electron, although some exist as marginally less reactive (albeit more diffusible) peroxide compounds. Table 1 categorises some of the biologically abundant species of reactive oxygen, their atomic composition, and their reduction potential as recorded by Halliwell and Gutteridge [14]. The two most stable and biologically relevant species are superoxide (O_2_^−^●) and hydrogen peroxide (H_2_O_2_), whose actions as signalling molecules will be the focus of this review.

Different measurement techniques exist that allow some scope for differentiation between ROS species and sources. In vitro plate-based assays such as the Amplex red-coupled horseradish peroxidase assay are commercially available and allow in vitro quantification of extracellular H_2_O_2_ levels [15]. Meanwhile, transgenic probes such as roGFP (1&2) and HyPer exhibit altered fluorescence when oxidised by intracellular H_2_O_2_ [16,17]. Specific intracellular sources of ROS may be probed by adding targeting sequences to HyPer and Timer in order to visualise mitochondrial, peroxisomal, or ER ROS production [17,18]. Perhaps the gold standard for detecting oxygen radicals is electron paramagnetic resonance (EPR), where the free electrons from radicals are stabilised on spin probes and absorb paramagnetic resonance. Since EPR is performed at cryogenic temperature, it does not provide a real-time measurement or information about subcellular localisation; however, the probes are cell permeable and offer a direct measurement of radical levels in cells and frozen tissue incubated with the spin probes [19]. Excitingly, real-time measurement of lung ROS has been performed via inhalation of EPR spin probes, helping to bridge the gap to real-time measurement [20]. With each of these techniques, it is possible to attempt to determine the respective levels of superoxide and H_2_O_2_ by adding combinations of superoxide dismutase and catalase, although this may interfere with the physiological cellular balance [21], especially considering that they may act as secondary messengers.

## 3. ROS as a Cellular Second Messenger

These ROS are often produced in response to various environmental or hormonal stimuli, which makes them prime candidates to act as cellular second messengers. By definition, and to function effectively, a second messenger must satisfy at least three main criteria:

- Firstly, in response to stimulation, a second messenger is able to swiftly propagate and amplify the signal intracellularly.
- Secondly, it must have downstream targets that trigger an effect.
- Finally, there must be a mechanism for efficiently ending the signal and effect within an appropriately short timespan.

For examples of how some “classic” second messengers fulfil these criteria, let us examine the function of cAMP and Ca^2+^. Following ligand binding to a G-protein-coupled receptor where the G-protein is G_αs_, adenylyl cyclase is specifically activated to rapidly increase [cAMP]_i_ as an amplifying second messenger molecule [13]. The cAMP then diffuses freely through the local milieu and instigates responses from multiple established targets, a canonical example being protein kinase A. Yet the [cAMP]_i_ signal is short-lived, being rapidly terminated by phosphodiesterases, which convert cAMP back to inert AMP within minutes [13]. Ca^2+^ behaves similarly. In response to stimuli, such as membrane depolarisation by a neurotransmitter, cytosolic [Ca^2+^]_i_ is quickly increased within seconds (either released from intracellular Ca^2+^ stores, such as the endoplasmic reticulum and the sarcolemma via ryanodine receptors (RyR), or via rapid intracellular influx through plasma membrane voltage-dependent Ca^2+^-channels) and may propagate further through Ca^2+^ induced Ca^2+^ release [22]. Ca^2+^ achieves its signalling effects by activating Ca^2+^-dependent targets such as conventional protein kinase C (PKC), calcium/calmodulin kinases, a variety of ion channels, and exocytotic proteins. Within minutes, ATP-powered Ca^2+^ pumps and Na^+^/Ca^2+^ exchangers then transport the Ca^2+^ back into the SR/ER, or out of the cell entirely, to rapidly terminate the signal [23].

In both examples, the short transient increase in [Ca^2+^]_i_ or [cAMP]_i_ might be considered a ‘biological switch’. Yet excessive production of either second messengers or compromised ability to terminate their signal leads to pathologies. This scenario might be considered analogous to that of “oxidative stress”, which occurs when ROS production chronically exceeds the levels at which it exerts beneficial effects. Not only does Ca^2+^ overload lead to calpain activation and cell death [24,25], but it also triggers mishandling of calcium in the mitochondria and subsequent energetic deficiencies, alongside pathologies such as cardiac arrhythmia in the context of cardiomyocytes [12,26]. There is increasing evidence that calcium overload in neurones, termed excitotoxicity, underlies neurodegenerative disorders such as Alzheimer’s and Parkinson’s diseases [27,28,29]. Meanwhile, cAMP overload in cardiomyocytes underlies diseases such as heart failure and ventricular tachycardia in humans [10,11,13,30].

Despite this, Ca^2+^ and cAMP are most famous for their signalling properties rather than their toxic actions when malfunctioning, while the signalling properties of ROS are perpetually overlooked. Instead, the metabolic disease field has tended to primarily focus upon the maleficent properties that ROS exhibit only when the system malfunctions, establishing them a sinister reputation. By considering the tightly regulated balance of ROS production versus ROS disposal, we emphasise below how ROS can demonstrate all three second messenger criteria and give documented examples of how ROS-S, when well controlled, enables good metabolic health.

### 3.1. Sources of ROS and Signal Amplification

Myriad ROS can be produced by an individual cell, with the type of species produced being specific to the source reaction and influenced by the cellular environment. Accordingly, individual cellular compartments have evolved to dispose of and/or propagate radicals they most commonly produce (highlighted in Supplementary Table S1). Since specific metabolic reactions are segregated and compartmentalised in the cell, so too are the enzymes comprising the ROS production machinery. The mitochondria, peroxisomes, cytosol, and endoplasmic reticulum are the main cell compartments that produce ROS, yet each does so through different means:

#### 3.1.1. The Mitochondria

**Figure 1:**
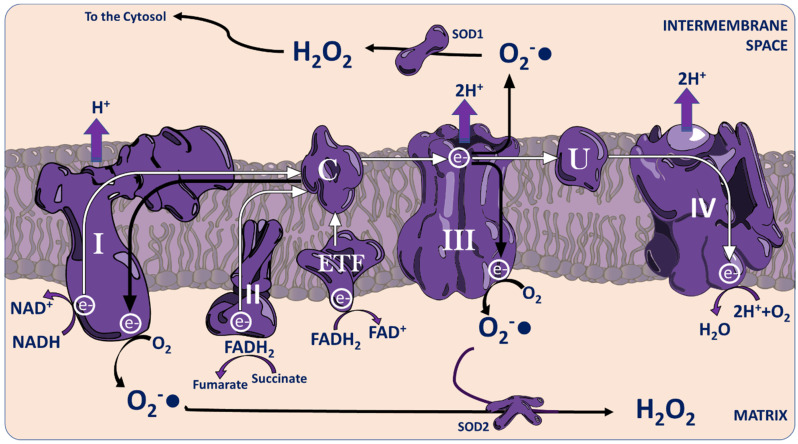
ROS Production at the Mitochondrial Electron Transport Chain. Electrons obtained from metabolic substrates in the form of NADH enter the electron transport chain (ETC) at complex I, while those obtained as FADH_2_ enter at complex II, or at the electron transferring flavoprotein (ETF) if their origin was extra-mitochondrial fat oxidation. In ATP synthesising “forward electron transfer”, those electrons are then transferred onwards to cytochrome c, complex III, ubiquinone, and complex IV in sequence, providing energy to pump protons into the intermembrane space. However, in conditions of stress or where oxygen is limiting, those electrons may be transferred to oxygen directly at complex III, generating the ROS superoxide either in the mitochondrial matrix or the intermembrane space (Black arrows). Additionally, electrons arriving at cytochrome c from complex II or the ETF may instead be transferred to complex I via “reverse electron transfer”, forming superoxide at complex I. Superoxide may then be converted to the more membrane diffusible H_2_O_2_ by Cu/Zn superoxide dismutase (SOD1) in the intermembrane space or Mn superoxide dismutase (SOD2) in the matrix.

Mitochondria hold a central role in oxidative metabolism and oxygen sensing [31]. At the mitochondrial electron transport chain (ETC), oxygen is combined with protons and electrons stripped from metabolites in the form of reducing intermediates (Figure 1). In physiological or diseased states, perturbation of the ratios in which these sub-atomic particles are combined with O_2_ yields ROS instead of the normoxic H_2_O end product. Complexes I and III of the ETC are the major complexes implicated in ROS production, with the most integral to oxygen sensing seeming to be complex III [32,33,34].

ROS production at the ETC also varies in correspondence to the ratio of metabolic substrates oxidised. Differing ratios of redox reducing equivalents FADH_2_ and NADH yielded by the catabolism of different metabolic substrates largely contribute to this variation. Physiological levels of glucose oxidation predominantly produce NADH. Per 5 NADH molecules, glucose oxidation yields only 1 FADH_2_, which exists only transiently at the ETC complex II. Meanwhile, β-oxidation of fatty acids yields more FADH_2_, which transfers electrons to the electron transferring flavoprotein (ETF) [35]. As a result, increased levels of fatty acid oxidation create greater competition to transfer electrons to ubiquinone. This is thought to drive both reverse transfer of electrons from the ETF via complex I to ROS and increased transfer to ROS via complex III, augmented by some ROS synthesis at the ETF itself [36,37,38,39,40,41]. On top of this, fatty acids consume more oxygen per gram of substrate oxidised than glucose [42,43,44,45], which further imbalances the ETC electron:oxygen ratio. It is possible that periods of greater FAO generate pulses of ROS that may signal to the rest of the cell about metabolic state and substrate availability.

#### 3.1.2. The Peroxisome

Peroxisomes, by their 1966 definition, are accountable for cellular fatty acid breakdown and are heavily involved in ROS metabolism [46]. The process of peroxisomal fatty acid metabolism by itself yields H_2_O_2_ in the first step [47]. Peroxisomal ability to produce, handle, and quench this ROS is expansive, yet perennially overlooked.

**Figure 2:**
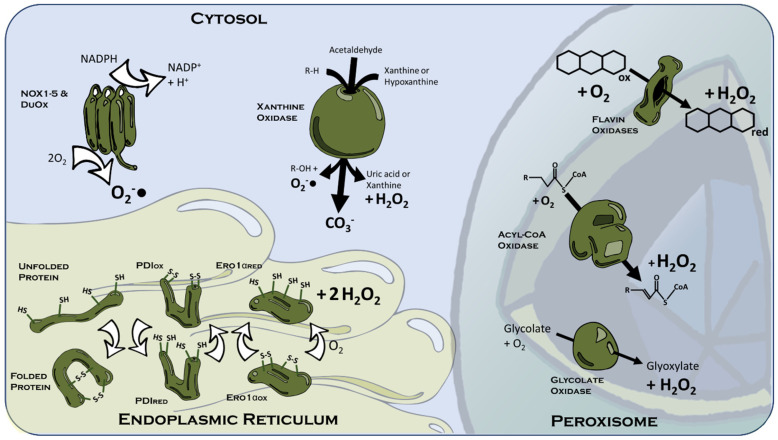
Common Cellular ROS Producing Enzymes. In the cytosol, NOX1-5 and the two dual oxidases (Duox) convert O_2_ to superoxide by hydrolysing NADPH. Xanthine oxidase may synthesise several species of ROS: superoxide is yielded from its oxidation of an alkyl R-H group to R-OH; CO_3_^−^● is produced from acetaldehyde, and hydrogen peroxide is generated from the transformation of xanthine or hypoxanthine to uric acid or xanthine, respectively. In the peroxisome, hydrogen peroxide is generated by the action of flavin oxidases, acyl-CoA oxidases, and glycolate oxidase. Meanwhile, in the endoplasmic reticulum, oxidised cysteine residues on protein disulphide isomerase (PDI) are reduced to provide the oxidising wherewithal for oxidation of cysteine residues on unfolded proteins. This allows the formation of disulphide bonds, which confer secondary and tertiary structure to folded proteins. PDI is then reoxidised by oxidised ERO-1α in a process yielding hydrogen peroxide as a product.

A multitude of enzymes make contributions to peroxisomal ROS generation (Figure 2). Glycolate oxidase (GOX) is one of the predominant players, converting glycolate and O_2_ to glyoxylate and H_2_O_2_ [48,49,50]. Acyl-CoA oxidases [47] and other flavin oxidases (xanthine oxidase, sulphite oxidase, sarcosine oxidase, diamine oxidase, and polyamine oxidase) each contribute further to peroxisomal H_2_O_2_ output [51,52,53,54,55]. Additional spontaneous and catalysed O_2_^−^● dismutation adds to the net product of H_2_O_2_. There are also a subset of superoxide generating enzymes in the peroxisome, with notable cameos for xanthine oxidase and certain peroxisomal membrane proteins [14].

Intriguingly, peroxisomes and mitochondria are often in proximity in the cell, allowing close communication [56]. PEX11, a peroxisomal membrane protein, and the mitochondrial protein MDM34 physically interact to help facilitate this, offering a potential conduit for signalling interaction [57]. Such signalling via ROS occurs, with H_2_O_2_ synthesised in the peroxisomes influencing both mitochondrial redox state and respiratory capacity [58,59,60]. Likewise, in mice with defective peroxisomal biogenesis, the mitochondria are impaired both morphologically and functionally, implying that peroxisome-mitochondria ROS-S is required to facilitate mitochondrial activity and, as such, overall cellular energy homeostasis [61]. It is therefore plausible that peroxisomal H_2_O_2_ is not necessarily confined to the peroxisomal matrix and may transmit messages to other cellular compartments.

#### 3.1.3. Cytosolic ROS Sources

Several ROS-producing enzymes are localised across the cytosolic compartment (Figure 2). NADPH Oxidase (NOX) comprises a discretely localised membrane-associated enzyme with its active site facing the cytosolic compartment of the cell. NOX oxidises NADPH and, less preferably, NADH to produce superoxide [62]. There are seven known mammalian isoforms of NOX, including the constitutively active NOX4, along with NOX1-3, which require cytosolic subunit association for function [63]. Meanwhile, NOX5 and the dual oxidase proteins (Duox1 and Duox2) are Ca^2+^ activated, signifying potential interplay with other cell signalling systems [64]. An example of this interplay occurs during thyroid induction of autophagy in the skeletal muscle [65]. NOX isoforms associate with specific membrane ruffles, lipid rafts, and caveolins, which facilitates the localisation of ROS-S in proximity to certain receptor-mediated signalling within the cell. For example, localisation of NOX4 with the AT1 GPCR in caveolin rafts has been shown to be essential for angiotensin II signalling and extends the concept that ROS handling enzymes are integral to certain cellular signalling machineries [66].

Another well-characterised cytosolic ROS-generating system involves xanthine oxidase (XO). XO is capable of producing several different varieties of ROS depending upon its substrate. In converting hypoxanthine to xanthine and xanthine to uric acid, XO generates H_2_O_2_ [67]. However, when oxidising a purine or amino acid attached hydrogen to an alcohol group, the enzyme generates superoxide [68]. Furthermore, XO has been documented to generate the highly reactive carbonate radical in the presence of bicarbonate [69]. The carbonate radical is an under-investigated radical in cellular biology, which persists intracellularly for a long period and has been suggested to underpin the ability of bicarbonate to oxidise methionine residues—thus also suggesting potential as a signalling molecule itself [70,71]. The variety of ROS generated by XO activity may signal important local information regarding the dynamic cellular metabolic environment.

#### 3.1.4. The Endoplasmic Reticulum

The Endoplasmic Reticulum (ER) is rather unique among cellular organelles in possessing an oxidative lumen [72]. Protein folding and maturation require oxidative reactions, such as hydroxylation of proline or disulphide bond formation between two cysteine residues. Protein disulphide isomerase (PDI) oxidises many of these cysteine residues in tandem with concurrent reduction of O_2_ to O_2_^−^● [73,74]. PDI activity is regenerated in turn through the production of H_2_O_2_ by the enzyme endoplasmic reticulum oxidoreductin 1 (ERO1), the human isoforms of which are ERO1α and pancreatic ERO1β [75,76]. Intriguingly, these enzymes may only scratch the surface of ER oxidative capacity, with viability and even a vestige of proinsulin biosynthesis (requisite of disulphide bond formation in the ER of endocrine pancreatic β-cells) retained in dual knockouts of both ERO1 isoforms [77]. However, rates of secretory pathway protein synthesis in a cell will be quite variable, and, as such, the oxidative capacity of the ER will have to be likewise adaptive. In the case of proinsulin biosynthesis, which can increase ≥50-fold via glucose-induced translational regulation [78], ER-oxidative capacity should be capable of adapting in parallel as previously shown [79]. Much is made of oxidative damage during diabetes, and antioxidant approaches are often proposed [80,81]. Yet inhibiting ER ROS production in pancreatic β-cells might actually exacerbate the disease, since (pro)insulin production could be compromised without appropriate ER-oxidative capacity present.

Alongside participation in secretory pathway protein synthesis, ER ROS products may also participate in ROS-S. The ER has been suggested to play in concert with the mitochondria and peroxisomes, together comprising a signalling composite that is in constant communication. ER membranes associate with the mitochondria to become mitochondria-associated membranes (MAM), and these points of contact are foci for ROS-S [82]. ERO1α localises almost exclusively to MAM contact points within the ER, as do multiple proteins with redox-sensitive cysteine residues (see Section 3.3 later), including, for example, the ryanodine receptor (RyR) responsible for mitochondrial calcium handling [82,83]. ROS-S at the MAM positively influences local mitochondrial calcium flux. When ERO1α is downregulated, mitochondrial calcium flux and respiratory capacity are impaired [83,84]. Similarly, ER contacts with peroxisomes may also be sites of ROS-S interchange between the two organelles. The ER closely contacts peroxisomes and can be found entwined around them [85].

### 3.2. Localisation and Termination of ROS Signalling

**Figure 3:**
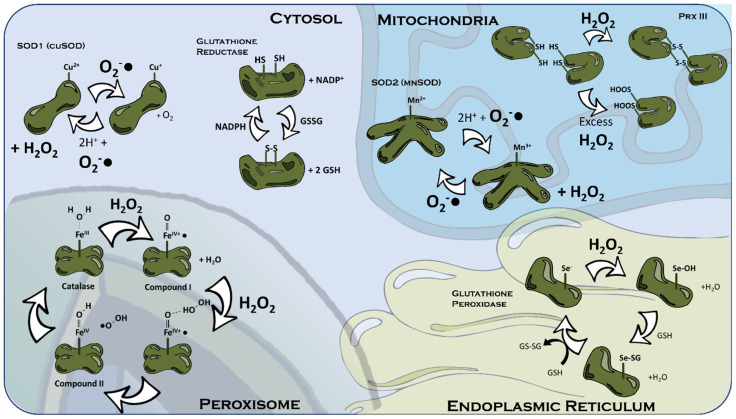
Common Cellular Antioxidant Enzymes. Both in the mitochondria and the cytosol, superoxide dismutase (SOD) 1 and 2 (containing Mn or Cu, respectively) convert two molecules of superoxide to hydrogen peroxide. Meanwhile, also in the mitochondria, peroxiredoxin III (Prx-III) contains two S-H groups, which are oxidised to disulphide bonds linking two PrxIII proteins by hydrogen peroxide. However, excess H_2_O_2_ inactivates Prx-III function by hyperoxidising its SH residues to SOOH. In the endoplasmic reticulum, the Se^−^ contained within glutathione peroxidase is oxidised to Se-OH by hydrogen peroxide and is then restored to Se^−^ by oxidation of two molecules of reduced glutathione (GSH) to oxidised, dimerised glutathione (GSSG). GSSG may be restored to two GSH monomers in the cytosol by oxidising two S-H groups on glutathione reductase to a disulphide bond, which are then reduced back to S-H groups by NADPH. The enzyme catalase is contained within peroxisomes, and the Fe^III^ it contains allows it to convert hydrogen peroxide to H_2_O in a cyclic process.

Once induced, the kinetics of secondary messengers are fast and transient, lasting only a few minutes, at most. The ability to turn off a secondary signal is just as important as the ability to switch them on. Increases in [cAMP]_i_ are well controlled by phosphodiesterases [13]. Likewise, rises in cytosolic [Ca^2+^]_i_ are transient and effectively reduced via uptake into the ER by sarco/endoplasmic reticulum Ca^2+^-ATPase (SERCA) [12]. An extensive enzyme system exists to dampen ROS signals in an analogous manner. NOX excepted, each aforementioned ROS source is localised within the hydrophobic membranes of specific organelles. This naturally limits the diffusion of ROS and keeps ROS-S spatially constrained. On top of this, each compartment possesses distinct enzymatic machinery for terminating the ROS signal. Given that species of reactive oxygen can be specific to a source reaction, each major cellular source of ROS possesses antioxidant enzymes tailored to the species of ROS produced there, the reactions of which are summarised in Figure 3.

**Mitochondria** possess multiple enzymes for degrading ETC-derived ROS, the most prevalent and best characterised being mitochondrial superoxide dismutase (MnSOD/SOD2) [86]. MnSOD is immediately on hand to handle the conversion of superoxide produced at the ETC to H_2_O_2_ [86]. An interesting question arises from MnSOD function because its H_2_O_2_ product still represents a ROS signal, and one with a longer range and lifespan at that. Therefore, is the purpose of MnSOD to terminate the signal or to transmute it into a more durable secondary messenger?

The mitochondrial outer membrane helps limit MnSOD-mediated production of H_2_O_2_ to the inter-membrane space, but mitochondria possess several additional mechanisms for localising the H_2_O_2_ they produce. Some examples of these are glutathione/glutathione peroxidase and peroxiredoxin III (Prx3) [87,88]. Glutathione peroxidase uses glutathione as an electron donor to convert H_2_O_2_ to H_2_O [87], although doubt has been cast over its significance by the report that knockout mice develop normally and appear just as capable of handling H_2_O_2_ challenges as wild-type animals [89]. Prx3 may be more crucial to intra-mitochondrial handling of H_2_O_2_. It displays two cysteine residues, which may be oxidised into cysteine bonds in order to dimerise it with another Prx3 and reduce H_2_O_2_ to H_2_O in the process [88]. This is effective only up until a certain concentration of H_2_O_2_ (~10 μM), concentrations exceeding which hyperoxidise and inactivate Prx3 [88].

As with the mitochondria, **peroxisomes** encapsulate their own antioxidant enzymes. Since most peroxisomal enzymes exude H_2_O_2_, it should not surprise that the dominant peroxisomal antioxidant is catalase [46]. These enzymes specialise in catalysing the degradation of H_2_O_2_ to water and oxygen via an oxidised intermediate known as catalase compound I [90]. Catalase was identified at the same time as the peroxisomes [46], and its presence is synonymous with these organelles. Meanwhile, peroxisomal superoxide is “defused” by peroxisomal variants of superoxide dismutase [53].

There is heterogeneity of peroxisomal catalase content, suggesting that some peroxisomes might play a greater H_2_O_2_ signalling role than others. Immunocytochemistry of density-separated rat liver peroxisome fractions found catalase-negative populations of peroxisomes that were instead enriched in acyl-CoA oxidase and peroxisomal multifunctional enzyme, while catalase itself seemed most prevalent in peroxisomes containing thiolase [91]. Acyl-CoA oxidase itself produces H_2_O_2_, so its localisation in catalase-negative peroxisomal subtypes could indicate that the ROS it produces is not necessarily pathogenic and instead involved in signalling. In the developing human liver [92], as well as in rat liver regenerating from partial hepatectomy [93], catalase-negative peroxisomes predominate, suggesting that ROS-S via H_2_O_2_ generation may be important for hepatocyte differentiation and/or liver regeneration. Further, in rats administered fibrate drugs (Peroxisome Proliferator Activated Receptor alpha (PPARα) agonists), hepatic peroxisome numbers increased dramatically but were mainly comprised of catalase-negative peroxisomes [94,95,96]. Intriguingly, in catalase knockout mice, livers displayed lower lipogenesis capacity, alongside a degree of protection against high-fat diet-induced steatosis and hyperglycaemia [97]. This suggests that peroxisomal H_2_O_2_ production could play a secondary signalling role in regulation of hepatic lipid metabolism, especially since little hepatic oxidative damage was observed in this model.

The **Endoplasmic Reticulum** (ER) contains a high concentration of glutathione, alongside an array of enzymes such as peroxiredoxin IV (Prx-IV), to tightly buffer its redox environment. Glutathione peroxidase catalyses the oxidation of a free cysteine residue on the ubiquitous reduced glutathione (GSH) by ROS [72,87]. This forms GSSG, two glutathione moieties connected by a disulphide bond, thus removing oxygen radical from the ER lumen. The ER variant of glutathione peroxidase is GPx8 [98]. Peroxiredoxin IV (Prx-IV) also preferentially localises to the ER and has been demonstrated to convert Ero1α-generated H_2_O_2_ to water and oxygen [99]. There are conflicting conclusions as to whether Prx-IV or GPx8 plays a greater role in ER H_2_O_2_ detoxification [98,99], although this is perhaps moot since both would terminate a potential ROS-S.

Finally, there are several cytosolic antioxidant systems, able to quench ROS escaping the confines of other organelles alongside that derived outside them. Chief amongst these are the cytosolic superoxide dismutase (Cu/ZnSOD, or SOD1) [86], and the glutathione couple [87]. Cu/ZnSOD, like MnSOD, facilitates the transformation of superoxide to two molecules of H_2_O_2_, so it may be a method of propagating an H_2_O_2_ signal as much as one that terminates the message of superoxide. Both systems are ubiquitous in the cytoplasm, increasing the likelihood of ROS being ‘deradicalised’ the further they travel from their source, and thereby helping constrain ROS-S spatially.

Additionally to these more abundant antioxidants, the **cytosol** also contains a variety of less abundant proteins that also serve to maintain redox homeostasis. Ferritin, an iron-containing protein, has been found to help convert H_2_O_2_ to H_2_O in the cytosol [100]. Meanwhile, thioredoxin assists in removing disulphide bonds on other proteins via the formation of an intramolecular disulphide bond between two of its own cysteine residues located on a catalytically available protrusion of its structure [101]. This assists in the termination of the effects of ROS.

### 3.3. ROS-S Transduction

Effective second messenger systems trigger specific downstream effects, and ROS can implement their signalling via direct effects on proteins and intermediary transcription factors. This concept of intracellular signalling through conformational changes in proteins that alter activity is well established in the context of protein phosphorylation. In that case, phosphorylation of a specific residue, a serine, threonine, and/or tyrosine, on an enzyme alters the conformation of the protein and affects its structure-specific catalytic ability [102]. ROS exhibit a similar capability to alter protein conformation via oxidation of certain accessible amino acids presented on the protein surface. Oxidation of specific cysteine residues to form disulphide bonds, either intramolecularly or to connect the residue with another molecule such as glutathione, can instigate a conformational change and consequently alter protein function. Methionines are also highly oxidisable amino acids, whilst arginine, proline, tryptophan, tyrosine, and lysine are amongst other amino acids known to be oxidised but less frequently [14,103]. ROS-mediated oxidation of these exposed residues in a protein may induce a physiological effect upon its activity.

As with phosphorylation, which occurs at conserved and enzymatically recognisable target sites upon an enzyme [102], certain sites on target proteins are particularly susceptible to oxidation. Cysteine residues are the best-established targets for oxidation, and their potential to be oxidised is influenced by their position in the molecule. Ferrer-Sueta and colleagues [104] give the example of Cys51 on human peroxiredoxin 2 (Prx-II), which may be oxidised much faster (k = 1 × 10^8^ M^−1^.s^−1^) than free cysteine (26 M^−1^.s^−1^) by H_2_O_2_. This reaction is oxidant specific—H_2_O_2_ alone benefits from enhanced PrxII reactivity [105]. The pKa value, or how readily a cysteine loses its proton to exist in an S^−^ state, correlates to reactivity with oxidants and is increased or decreased depending upon the electronegativity of adjacent amino acid side chains [104]. However, pKa alone does not account for specificity such as PrxII Cys51’s selectivity for H_2_O_2_, which may instead be determined by the surrounding structure. In particular, two factors enable oxidant specificity of an accessible cysteine residue: the ability of the surrounding residues to stabilise the oxidant-specific anionic transition state and to control access to the active cysteine by selecting for the shape and polarity of the oxidant [104,106]. Thiol residues may be oxidised to sulphenic acid (-SOH) and then subsequently to sulphinic (-S(O)OH) and sulphonic (-S(O)_2_OH) acids depending upon how oxidative the environment is [107,108]. Methionine oxidation to become MeSO also exerts signalling effects, and control of methionine oxidation may be regulated similarly by residues adjoining the oxidation site [109].

**Figure 4:**
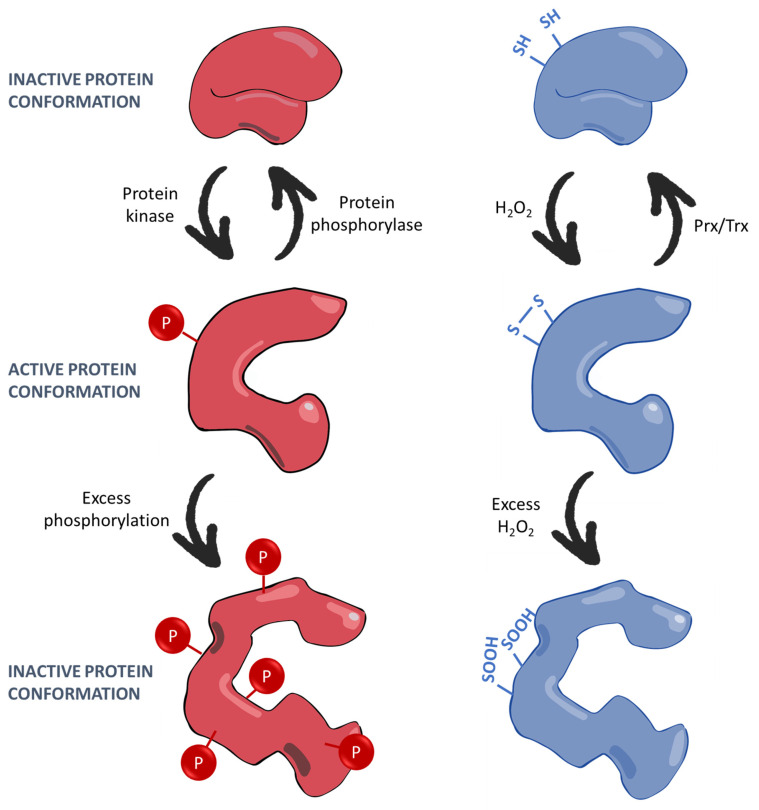
Oxidation of Amino Acid Residues Modulates Protein Activity in an Analogous Manner to Phosphorylation. Proteins activated by phosphorylation (red) exist inactive in their non-phosphorylated state, similarly to how oxidation-activated proteins (blue) exist inactive while their oxidisable residues remain in the reduced form. Both phosphorylation and oxidation of these respective residues can cause a change in the protein’s conformational shape, activating the proteins to perform their individual cellular functions. Meanwhile, in pathological environments, both over-phosphorylation and over-oxidation of the target residues may cause further, less beneficial alterations to the active site, impinging upon or triggering inappropriate protein function.

Just as protein phosphatases terminate phosphorylation-mediated signals by removing phosphate moieties from phosphoproteins, so proteins such as the aforementioned thioredoxin are able to terminate redox-related signals by reducing oxidised protein residues (Figure 4). As an example, thioredoxin 1 and thioredoxin reductase 1 have been shown to reverse the oxidation of protein tyrosine phosphatase 1b (PTP1B) by H_2_O_2_, thereby helping terminate the cyclical redox signals of growth factors [110]. Furthermore, later in the “Circadian Metabolism” section, we shall encounter sulfiredoxin, which terminates circadian redox-mediated inactivation of PrxIII by reducing H_2_O_2_-oxidised cysteine residues on the protein [111]. Such mechanisms provide “off switches” for ROS-S, necessary to maintain the signal as a physiological and beneficial one and prevent it from becoming “oxidative stress”.

A relatively well-characterised protein regulated by cysteine oxidation is hypoxia-inducible factor 1α (HIF-1α), which is commonly considered a sensor of cellular oxygen levels [112]. The protein can “sense” falling partial oxygen pressure by directly detecting resultant changes in mitochondrial ROS production [34,113,114]. Under normoxic conditions, the enzyme prolyl hydroxylase targets HIF-1α for von-Hippel Lindau factor-mediated degradation in the proteasome [115,116], yet in conditions of lower oxygen availability, the ratio of electrons:O_2_ available at the ETC increases. The result is increased superoxide production, which in turn inhibits prolyl hydroxylase, seemingly through oxidation of cysteine residues [117]. In prolyl hydroxylase’s absence, HIF remains active in the cell and effects metabolic changes in response to ROS production. Such O_2_-sensing via ROS production enables protective metabolic adaptation to otherwise damaging pathophysiological environments. In the heart, a drop in O_2_ and resultant increased ROS levels can drive a HIF-mediated shift from oxygen-intensive fatty acid oxidation (FAO) towards glucose oxidation, potentially aiding a failing heart unable to supply itself with enough oxygen [118,119]. Once high ROS levels have helped to stabilise HIF, it brings about this shift away from FAO by preventing transcription of PPARAα, a master upregulator of fat metabolism [120]. Likewise, at high altitude, ROS is produced at the ETC due to lower O_2_ levels, activating HIF and inducing erythropoiesis to enable greater red blood cell production [121].

ROS-S modification of many other proteins exerts control over metabolic pathways. Broad-acting phosphatases and kinases are common targets for cysteine oxidation, demonstrating a capacity for ROS-S to interface with other signalling cascades and further amplify its signal [122,123,124]. The 5′ adenosine monophosphate kinase (AMPK) is pivotal to responding to many metabolic scenarios, largely to switch to a more catabolic state and mobilise internal fuel stores when cellular energy is low. AMPK is metabolically regulated not only by AMP and its phosphorylation state but also by stimulatory hydrogen peroxide oxidation of cysteine residues [125]. Similarly, oxidation regulates mitogen-activated protein kinases (MAPK) and their phosphatases [126,127], which are involved in the H_2_O_2_-dependent relay of growth factor signalling [128]. Additionally, metabolic enzymes, including creatine kinase and glyceraldehyde-3-phosphate dehydrogenase [129,130], feature heavily as direct recipients of ROS-mediated cysteine oxidation, perhaps unsurprisingly given the links between metabolism and oxygen demand. Pyruvate kinase M2, for instance, which catalyses the final step of glycolysis, is inhibited by oxidation of Cys358 [131]. This limits oxygen-consumptive mitochondrial glucose oxidation under conditions of oxygen limitation. Similarly, creatine kinase, which regenerates ADP for oxidative phosphorylation by transferring a high-energy phosphate from ATP to phosphocreatine, facilitates greater oxygen consumption at the ETC. Its inhibition through the oxidative formation of a disulphide bond between Cys74 and Cys146 may therefore have an oxygen-sparing effect [132].

Thus far, a gap of knowledge has existed for ROS-S in that relatively few specific Cys and/or Met oxidation sites on proteins have been catalogued, in contrast to the many Ser/Thr/Tyr protein phosphorylation sites identified as part of protein kinase signal transduction cascades. This, in part, has been due to a deficiency and technical difficulties in being able to measure specific protein oxidation. However, with the advent of novel proteomic technologies able to better detect post-translational modifications (PTMs) of proteins, an era of being able to better identify specific Cys/Met protein oxidations is emerging that should then enable a much better appreciation, understanding, and acceptance of the importance of ROS-S.

### 3.4. Localised Signalling vs. “Oxidative Stress”—A Matter of Magnitude?

We have seen that ROS-S occurs and is localised in the vicinity of the origin organelle by organelle-specific antioxidants. So, too, have we seen reported many times that a cell-wide ROS burst may have larger, catastrophic consequences. It seems, therefore, that the threshold for these larger events, the point at which ROS-S crosses from that considered physiological to that considered pathological, must occur when local antioxidant capacity is exceeded and overwhelmed.

There are analogous scenarios with other second messengers. For example, calcium signalling occurs constantly in the heart and muscle as low-level signals termed “sparks”, which do not trigger cell-wide depolarisation [133]. Calcium sparks convey a multitude of messages, including opening Ca^2+^ activated K^+^ channels to hyperpolarise and relax the cell [134,135]. Sparks, as a localised signal, thereby demonstrate a completely opposing effect to larger spikes in Ca^2+^ which induces calcium-induced calcium release and contraction of the cell [133].

Overwhelming bursts of ROS, often supplemented by ROS-induced ROS release, also often differ from localised ROS-S in terms of physiological response [136]. One example is the low-level production of ROS at the mitochondrial level, which induces the turnover and regeneration of older mitochondria [137,138,139]. Mitochondria have a limited lifespan mostly due to the reactive nature of the intermediates they handle, and when they begin to show signs of wear, it is important to replace them. Damaged ETC components are often hallmarked by increased production of ROS. Mitochondrial autophagy and biogenesis are then brought about through a multitude of localised ROS-S cascades, including HIF activation [140,141]. An example is H_2_O_2_-mediated oxidation of Kelch-like ECH-associated protein 1 (KEAP-1) that alleviates its inhibition on the transcription factor. Nuclear factor-erythroid factor 2-related factor 2 (NRF2), allowing NRF2 to translocate to the nucleus and coordinate with the transcriptional co-activator, peroxisome proliferator-activated receptor gamma coactivator 1α (PGC-1α), to drive mitochondrial biogenesis [142]. The more localised ROS-S in this scenario thereby results in a beneficial physiological signalling effect.

Conversely, in scenarios such as cardiac and renal ischaemia reperfusion (I/R) injury, where oxygen supply is almost completely attenuated, the electron:O_2_ ratio at the ETC becomes greatly increased, and a much larger and longer ROS burst results [143]. This chronic reactive intermediate signalling (ChRIS) can overwhelm the mitochondrial antioxidant capacity and amplify itself in other areas of the cell by stimulating the likes of NOX to enjoin in ROS-induced ROS release [136]. The result is a completely opposing effect on cell viability—rather than rejuvenating the mitochondrial population like localised ROS-S; ChRIS signals for controlled cell death; eventually acting on caspase enzymes to induce apoptosis [144].

## 4. Examples of Physiological ROS-S Systems Relevant to Metabolic Disease

ROS-S systems exist throughout metabolism and can generally be characterised by identifying the following components:

- How does the putative agonist or physiological situation stimulate ROS production?
- What is the cellular source of the signalling ROS?
- What are the physiological targets of the ROS signal?
- What are the ROS-S system’s functional effects?

Below, five established metabolism-related ROS-S systems are explored to give selected examples of how ROS-S is harnessed for useful purposes in physiology.

### 4.1. Muscular Glucose Uptake and Metabolism

Even mild muscle contraction engenders ROS production [145], leading some to speculate that ROS-S is a mechanism for cell signalling during exercise. With enhanced oxygen demand during exercise, muscles’ mitochondria become oxygen-limited, and the ETC produces ROS, which could be the stimuli that differentiate exercise-induced from insulin-induced glucose uptake. Increased production of H_2_O_2_ was found to enhance glucose uptake in L6 myotubes [146] and in various muscle tissues from lean Zucker rats [147]. Overexpression of MnSOD in mouse musculature enhanced contraction-mediated glucose uptake by 25%, whilst the glutathione peroxidase mimetic, Ebselen, inhibited the same function [148]. Together, this indicates H_2_O_2_ rather than superoxide-mediated signalling. Supplementation of N-acetyl-cysteine (NAC) as an antioxidant cuts contractile enhancement of glucose uptake by half and decreases AMPK phosphorylation by a similar margin [148]. This tallied with earlier research reporting that both H_2_O_2_ and muscle contraction activated AMPK, and that this occurred via the AMPKα isoform and independently of AMP/ATP ratios [149,150]. AMPK activation with the agonists AICAR and Metformin has also been shown to increase cell surface expression of glucose uptake transporters 1 and 4 (GLUT 1 and 4) [151]. AMPK is known to be activated by H_2_O_2_, probably via oxidation of cysteine 299 and cysteine 304 since mutation of these residues to alanine abrogated the enhancing effect of H_2_O_2_ on kinase activity [125]. It, therefore, seems quite likely that contraction-induced ROS-S activates AMPK by oxidation, thus mediating the enhanced glucose uptake, and could partly account for poor results obtained for supposed ‘muscle supplementation’ with antioxidants.

### 4.2. Muscular and Myocardial Contractility

Alongside its effects upon glucose metabolism, ROS-S is integral to muscle contraction. In muscle, heart, and the vasculature, the presence of ROS is requisite for the greatest force to be achieved [152,153,154]. This is a further case of interaction between calcium signalling and ROS-S because muscle contraction depends upon RyRs being activated by cytosolic calcium, then opening and releasing further calcium from the sarcoplasmic reticulum into the cytosol. This calcium then binds and conformationally alters the muscle fibre protein troponin, thus allowing actin filaments to bind myosin and drive contraction of the muscle. The core function of ROS in muscle contraction appears to stem from a calcium-sensitising action, with oxidation and glutathionylation of RyR cysteine residues mediating increased calcium release and contractility [154,155,156]. Treatment of non-fatigued muscle with oxidants enhanced the contractile capability of fibres [152,153,157]. Meanwhile, supplementation with “antioxidants” had the opposite effect, yet another example of certain findings countering the dogma that ROS are always pathological and antioxidants are good [152,157,158,159]. The source of ROS in exercising muscle is easy to conjecture, as increased O_2_ demand during exercise leads to enhanced mitochondrial generation of ROS. Only in fatigued muscle, where ROS production has exceeded antioxidant activity and ChRIS occurs, do antioxidant treatments have a beneficial effect on contraction [159,160].

Certain endocrine agonists employ ROS-S to enhance cardiac contractility. Endothelin 1 is such an agonist [161]. Its effect is mediated by ET(A), but not ET(B), receptors and stimulates a burst of superoxide alongside enhanced L-type calcium channel opening [162]. L-type calcium channels in the cell membrane permit inward calcium currents, which subsequently stimulate RyR opening and cardiomyocyte contraction. Tempol (a ROS scavenger), SOD, and a NOX inhibitor each abolished the endothelin-stimulated enhancement of L-type calcium channel sensitivity, demonstrating that the ROS burst is integral to its signalling effect [162]. It seems, therefore, that endothelin 1 must stimulate NOX, and it would be interesting to know whether ET(A) is also localised in NOX-containing lipid rafts. Given the aforementioned oxidation of RyR receptors [154,155,156], it seems reasonable to hypothesise that ET(A) signalling may also induce RyR oxidation and enhanced release of calcium, which in turn stimulates enhanced cardiomyocyte contractility.

### 4.3. Angiogenesis

Both in the revascularisation of ischaemic tissue and in exercise-induced muscle growth, a dearth of oxygen induces vasoproliferation [163,164]. This effect is replicated by ROS, including physiologically by macrophages when superoxide bursts stimulate angiogenesis in an antioxidant antagonisable manner [165,166]. On top of direct action, peptide messengers such as Angiotensin II (AngII), angiopoietin I, VEGF, and catecholamines can stimulate angiogenesis via ROS intermediates [167,168,169,170,171]. Mueller et al. identified a novel redox sensor, Id3, which was essential to AngII-mediated angiogenesis [172]. Since catalase did not abrogate this effect, and superoxide dismutase did despite its H_2_O_2_ product, it appears that the second messenger involved in this cascade is likely superoxide. Induced xanthine oxidase production of superoxide also stimulated angiogenesis [172]. This superoxide signalling seems common to all peptide stimulators of angiogenesis, and the signal is antagonised when gp91^phox^ (a subunit of NOX) is knocked down [173]. Since NOX associates with the angiotensin AT-I receptor in lipid rafts [66], it is probable that AT-I receptor agonism activates NOX, generating superoxide signal transduction, perhaps via a mechanism analogous to GPCR signalling or via membrane depolarisation as previously documented in the vasculature [174,175].

AngII/NOX-mediated angiogenesis is also an excellent example of interplay between ROS signalling from multiple sources. PDI migration from the ER is essential to NOX activity [176,177], and PDI overexpression in vascular smooth muscle cells (VSMCs) enhanced basal superoxide production threefold—although this had the consequence of preventing AngII from further increasing NOX activity [178]. Meanwhile, excessive mitochondrial ROS production instigated by ethidium bromide or antimycin A also lowered the capacity for AngII activation of NOX and concomitant angiogenesis [179]. This suggests that localisation of the redox signalling is likely an important go/no-go decision influencer in angiogenesis, and if VSMCs are damaged or under global metabolic stress, they are conditioned not to participate in vascularisation. Excessively high levels of cytosolic ROS could inactivate NOX, perhaps via greater oxidative modification. In normal NOX functionality, this mechanism could also serve as feedback to shut down enzyme activity once sufficient ROS has been produced, and under conditions of oxidative stress, it could serve to suppress the proliferation of unhealthy/dysfunctional cells.

### 4.4. Glucose and Sodium Resorption in the Kidney

One of the primary functions of the kidney is the retention of sodium and glucose in the blood to regulate water retention and osmolarity. Following high-pressure blood filtration at the kidney’s glomeruli capillary beds, fluid undergoes phased active sodium and water resorption [180]. Initially, around 60–70% of sodium ions are reabsorbed in the proximal convoluted tubule, followed by osmotic resorption of H_2_O [180]. In the water-impermeable thick ascending limb of the loop of Henle, 20 to 30% of the filtered NaCl is then absorbed via Na^+^/2Cl^−^/K^+^ cotransporter across a gradient generated by the basolateral Na^+^/K^+^ ATPase [180]. This generates an osmotic gradient, which allows later retention of water from the filtered fluid. To maintain homeostatic blood pressure and water availability, this process is tightly regulated by various signalling processes, including ROS-S (Figure 4).

With regards to NaCl resorption, it has been demonstrated that superoxide stimulates the thick ascending limb of the loop of Henle (TALH) uptake of sodium, a process nitric oxide (NO) inhibits [181,182,183]. Through the usage of catalase and the SOD mimetic Tempol in tandem, this action upon NaCl resorption was shown to be mediated by superoxide but not H_2_O_2_. TALH superoxide is produced by NOX in response to physiologically increased lumen NaCl concentrations [184], although it is thus far unclear exactly how increased NaCl induces NOX activity. It is possible that increased movement of Na^+^ and Cl^−^ ions across the cell membrane leads to depolarisation [185,186], which also links to NOX activation in the separate setting of vasoconstriction [174,175], but requires further investigation in the TALH. Identical effects of ROS-S mediated increased NaCl uptake are observed in response to increased luminal flow rate and response to high luminal glucose concentrations [187,188,189,190,191,192]. It could be that stretching of the cell membrane instigates this depolarisation, while glucose uptake both alters osmolarity and provides a source of ATP to drive cross-membrane ion transport.

Downstream of NOX, the superoxide produced acts via protein kinase C alpha (PKC-α) to enhance the rate of ion uptake by the Na^+^/K^+^/2Cl^−^ cotransporter [193,194]. Inhibition of PKC ablates superoxide-mediated NaCl resorption [192], and conventional PKC isoforms have previously been demonstrated to be activated by ROS [195]. Activation of certain conventional, novel, and atypical PKC isoforms has been shown to occur through oxidation of the kinase’s cysteine-rich zinc finger region, which causes release of zinc and subsequent instigation of kinase activity independently of DAG or calcium [196]. When active, PKC-α phosphorylates the N-terminal region of the Na^+^/K^+^/2Cl^−^ cotransporter [197]. Phosphorylation of this region increases ion transport, while kinase inhibition halves the ion transportation rate in HEK 293 cells [197]. The resulting retention of NaCl in the TALH increases the osmolarity of the fluid remaining in the lumen and therefore facilitates greater water retention via osmosis at a later stage of the nephron [180]. ROS-S mediated by superoxide in the TALH therefore plays an important role in homeostasis and could be crucial in preventing hypotension/dehydration.

**Figure 5:**
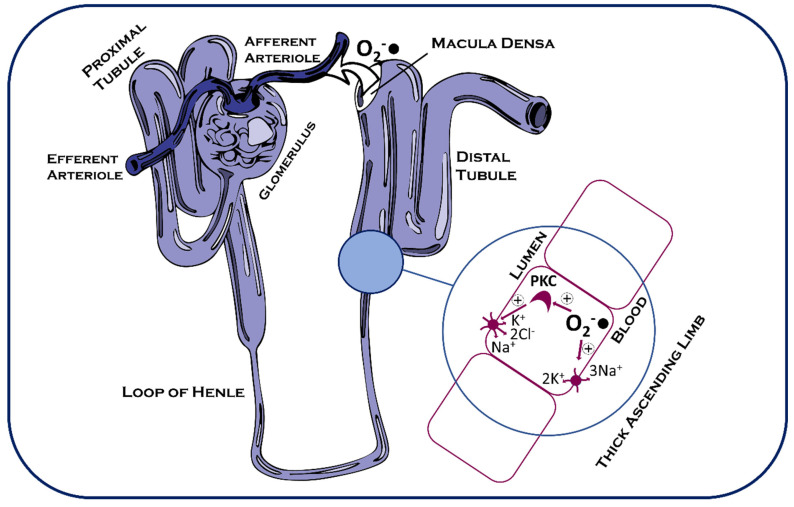
ROS-S in the Nephron. Superoxide produced at the macula densa in response to NaCl induces afferent arteriole constriction, restricting blood flow to the glomerulus. Meanwhile, in the thick ascending limb of the loop of Henle, superoxide activates protein kinase c (PKC), which subsequently increases NaCl uptake from the lumen. Superoxide also stimulates basolateral Na^+^/K^+^ ATPases to further augment the process of NaCl and water resorption.

Nephron-derived ROS also exhibits the capacity to act in a paracrine manner upon other parts of the kidney. The macula densa sits at the top of the TALH, close in proximity to the glomerulus and afferent arterioles (Figure 5). Macula densa cells detect NaCl concentration changes in the tubular fluid and feedback upon afferent arteriole vasoconstriction, thus influencing the pressure at which blood is filtered at the glomerulus. This feedback loop is known as tubuloglomerular feedback, and ROS-S has emerged as a critical signal involved in this mechanism [198,199]. Macula densa depolarisation occurs in response to altered transmembrane ion movement when luminal NaCl alters, and this triggers superoxide production [186]. While both NOX2 and NOX4 are expressed in the macula densa, it is NOX2 that is primarily responsible for NaCl-induced superoxide production, and this is mediated by the translocation to the membrane of the Rac GTPase [186,200]. Rac hydrolyses GTP for energy, and NOX1-3 are dependent upon its function [201]. The resultant superoxide then diffuses from the macula densa and stimulates afferent arteriole constriction in dual-perfused setups. The target proteins oxidised in the afferent arteriole have not yet been identified, but given the enhancement of smooth muscle contraction involved, it seems reasonable to assume that known Ca^2+^ handling targets of ROS-S, such as the RyR, would be decent candidates to investigate. The O_2_^−^● also performs a further function in terms of reacting with the established vasodilatory agent NO to form NOO-, effectively scavenging the vasodilatory signal generated by falling luminal NaCl [202].

### 4.5. Circadian Metabolism

For most higher organisms, any 24 h period is composed of regular periods of inactivity (sleep) and periods of activity. Activity corresponds to energy requirements, and therefore this cyclic aspect of life is reflected in the metabolic generation of ATP. Increased metabolism and oxygen utilisation during the active period also result in increased generation of mitochondrial H_2_O_2_, and it has become apparent that these redox cycles are influential in the maintenance of biological rhythms [203]. At certain times of the day, H_2_O_2_ production occurs in sufficient magnitude to purposefully oxidise the mitochondrial PrxIII enzymes, allowing it to escape into the cytosol and function as a circadian signalling component [204].

The adrenal cortex is one organ where circadian ROS-S has been indicated. PrxIII is the most highly expressed antioxidant enzyme in the cortex, and its inactivation by excess H_2_O_2_ allows further H_2_O_2_ to escape from the mitochondria and activate p38 MAPK [205]. The p38 MAPK inhibits Steroidogenic Acute Regulatory protein (StAR—the key regulatory protein in steroid hormone synthesis that enhances cholesterol to pregnenolone production), and thereby the adrenal generation of corticosterone alongside its daughter hormone aldosterone. Corticosterone has weak glucocorticoid properties, meaning that it promotes gluconeogenesis and inhibits glucose uptake, while aldosterone inhibits glucose uptake (also involving a ROS-S contribution) alongside enhancement of renal sodium and water potential [206,207]. During the resting phase of the circadian cycle, therefore, ROS-S allows the adrenal gland to help conserve energy stores for use during the active phase. The effectiveness of this cyclical signalling is preserved by cytoplasmic H_2_O_2_ acting to terminate its own message. Hyperoxidised PrxIII in the mitochondria is restored to the reduced form by a protein named sulfiredoxin, and this, in turn, may only enter the mitochondria as part of a disulphide-linked complex with heat shock protein 90 [111]. H_2_O_2_ arriving in the cytoplasm may catalyse the formation of this bond, and with the end of the active period, the reduction of PrxIII by sulfiredoxin comes to outweigh the hyperoxidisation of PrxIII by H_2_O_2_, thus allowing a return to corticosterone synthesis [111,205]. Not only is this an example of H_2_O_2_ catalysing the formation of a disulphide bond to transduce a cellular message, but it is also an excellent example of how ROS-S signals are terminated once their message has been received.

### 4.6. Aging Metabolism

Ageing constitutes one scenario where evidence suggests excessive reactive oxygen species production damages cells. The free radical theory of ageing, initially put forward by Harman, suggests a sustained increase in ROS production and a decrease in enzyme capacity for terminating the ROS signal [208,209]. This leads to a situation where ChRIS persists, disrupting normal ROS-S and damaging cellular membranes, alongside proteins by peroxidation. An example of this in ageing is the mitochondria-ER contact sites, whereby ageing-induced ROS from the ER stimulates further excessive mitochondrial ROS production and disrupts calcium signalling [210]. The situation is not so simple as ROS directly causing ageing, however—instead; evidence demonstrates that ROS can also work to delay the ageing process.

More transient ROS signals have been demonstrated to upregulate the cellular ROS control enzymes in a process termed mitohormesis [211,212,213]. Without ROS, therefore, the ageing process would be accelerated. This is also evident in exercise training, where ROS produced during exercise actually prolongs lifespan, even to the extent that the protective effects of exercise-induced ROS are still observed in offspring [19]. This is potentially activated by the NRF2/KEAP1 signalling pathway, among others. KEAP1 has redox-sensitive cysteines, which sequester the NRF2 transcription factor until ROS signals for its release, thereby activating a variety of anti-ageing signalling pathways [214,215]. There is a further argument that increased ChRIS in older age is caused not by over-production of ROS, but by lack of physical or mental activity to consume the products of metabolism [216]. This then leads to downregulation of metabolic capacity and subsequent inability to respond to metabolic challenges [216].

## 5. “Oxidative Stress”—Where Heroes Become Villains?

Inevitably, as with excesses of Ca^2+^ and cAMP, excessive ChRIS may become toxic to cells and tissues. Chronically elevated concentrations of reactive oxygen intermediates become indiscriminate in the proteins they oxidise. Oxidation of proteins and other molecular entities such as membrane lipids rises, and this can then interfere with cellular processes, as well as activate caspases to induce apoptosis. However, further work is needed to clarify the exact contribution of excessive ROS to the progression of metabolic disease.

Due to the overabundance of metabolic substrates present in the setting of metabolic disease, scenarios, where surfeits of fatty acids or glucose may directly lead to pathology, are often thought about. Those who suggest that these environments are toxic have dubbed them “Lipotoxicity” and “Glucotoxicity” respectively [217,218], and in combination, “Glucolipotoxicity” [219]. These scenarios are hypothesised to arise in disorders including type 2 diabetes, metabolic dysfunction-associated steatotic liver disease (MASLD)/hepatic steatosis (MASH), diabetic kidney disease (DKD), and heart failure (HF), where they would engender ROS production, which mediates disease progression. The premise is sound, as it is certainly the case that overload of other secondary messengers underlies pathology [11,13]. Despite this, there has not yet been a convincing demonstration of a mechanism for “oxidative stress” at the molecular level.

Greater levels of fatty acid oxidation and/or glycolytic metabolic flux leading to increased flux through the mitochondrial ETC (and/or peroxisomal fatty acid breakdown) would be expected to yield more ROS. However, molecular mechanisms for how oxidative cellular damage could result from such chronically elevated metabolic flux remain poorly defined. Moreover, how such a cellular injury would be recognised by the immune system to trigger an inflammatory response and subsequent fibrosis (perhaps in a vain attempt to repair the damage) remains to be elucidated. Are abnormally oxidised proteins recognised as ‘neoantigens’ that attract a targeted immune response? It is also worth noting that some organs, including the heart, kidney, and liver, utilise almost exclusively fatty acids for ATP generation under some physiological conditions [220,221,222,223,224], suggesting that they are more than capable of coping with ROS production associated with high levels of lipid oxidation. Due to this, correlating increased cellular lipids with greater ROS levels is not necessarily enough to conclude causation for “oxidative stress”.

Carefully constructed experiments will be required to definitively demonstrate a role for “oxidative stress” in chronic metabolic disease if we are to avoid the assumption that all ROS are bad ROS. Firstly, the exact mechanism by which excessive metabolic fuel (lipid in particular) overload leads to ROS production must be defined—is it mitochondrial; and if so; from which complex? Alternatively, could it be peroxisomal due to an increase in long-chain lipid oxidation? Or NOX-related, perhaps due to alterations in membrane lipid composition? Crucially, it must be demonstrated that this ROS overload is outwith the range of normal physiological ROS-S and is not merely an adaptive signal (e.g., to stimulate increased FAO). The identification of specific Cys/Met oxidation sites on proteins could be pursued, and a distinction made between those that are normally oxidised as part of the ROS-S mechanism and those that are abnormally oxidised as a consequence of ChRIS leading to cellular dysfunction. Following this, elucidation of a solid mechanism by which chronic metabolically induced ROS triggers the progression of metabolic disease pathology in organs such as the liver, kidney, and heart would be required.

Considering MASLD/MASH pathogenesis as an example, certainly ROS does not cause intracellular lipid accumulation, which is the first hallmark of the disease since a case is being made that it is heightened hepatic lipid oxidation that triggers chronic ROS production. So, it must be demonstrated that ROS causes the second hallmark of diseases like MASLD/MASH—inflammation; fibrosis; and cell death. This is possible, for ROS is known to activate caspases when present in excess [144]. Yet there are many other aspects of the MASLD/MASH environment that are equally, if not more, likely to be responsible for fibrosis, including the fatty environment of the liver leading macrophages to become “foam cells” in an analogous manner to atherosclerosis [225,226]. Until it is convincingly demonstrated that without chronic ROS production, MASLD/MASH progression is markedly delayed, a conclusion that hepatic ROS production (associated with greater levels of lipid metabolism) is a major contributor to disease pathogenesis cannot be conclusively made. If such mechanistic insight could be gained, it would also then be important to determine the concentration threshold above which ROS production ceases to be physiological and becomes pathophysiological.

While some have been identified, a complete list of oxidisable proteins involved in ROS-S is far from established. Once these have been definitively characterised (a characterisation that will most likely depend upon the cell type), it will become easier to identify proteins oxidised during ChRIS, which are not necessarily involved in ROS-S. Not only will this allow a thorough investigation of physiological processes that may utilise ROS-S, but it will also enable a better understanding of ROS-associated pathological processes, how these differ from ROS-S, and whether they are specific to different disease states.

## 6. Closing Insights

General misconception abounds that ROS has no function beyond being toxic harbingers of cellular dysfunction, death, and destruction. Rather than this negative outlook, which is perhaps over-represented in the literature, this review outlines how ROS may more commonly act as second messengers of an important and tightly regulated system.

The normal tightly controlled ROS system may malfunction, and in disease states such as diabetes and MASH, this malfunction may contribute to pathology. Despite this, attempts to attenuate ROS production therapeutically are likely destined to be unsuccessful—even if the ultimate “antioxidant” were discovered, it might well interfere with crucial homeostatic signalling. A prime example of this is presented by the ubiquity of ROS-S within glucose homeostasis: abolition of ROS in a diabetic setting may well instigate the very inability to fully regulate glucose metabolism that defines the disease itself. Attempted antioxidant treatment would also impair cardiac contractility, not a side effect to be taken lightly when diabetics represent 40% of heart failure sufferers [227]. For these reasons and more, as a scientific community, we might better consider and explore ways to work with ROS-S to combat metabolic disease, rather than making it the enemy.

## Figures and Tables

**Table 1:** Biologically Prevalent Reactive Oxygen Species.

ROS	Chemical Composition	Reduction Potential (V)
Oxygen	O_2_	−0.35
Superoxide	O_2_^−^●	0.94
Hydroxyl radical	OH●	2.31
Hydrogen peroxide	H_2_O_2_	0.32
Carbonate radical	CO_3_^−^●	1.78
Alkoxyl radical	R-O●	1.60

## Supplementary Materials

The following supporting information can be downloaded at: https://www.mdpi.com/article/10.3390/ijms26062622/s1.
