# Supplementary material for: Reactive Oxygen Species (ROS) in Metabolic Disease—Don’t Shoot the Metabolic Messenger
Source: Int J Mol Sci. 2025 Mar 14;26(6):2622. doi: 10.3390/ijms26062622 (PMC11942130; doi:10.3390/ijms26062622)
Supplement: Supplementary file 1 [file ijms-26-02622-s001.zip › ijms-3480115-supplementary.pdf]

Supplementary Table S1: An outline of the majority of ROS generation, propagation and termination enzyme expressed in human cells.

Enzyme	Abbreviation	IUBMB EC number	Function	Intracellular Localisation	Human Tissue Expression
**ROS Sources**					
**NADPH Oxidase 1-5**	NOX1-5	EC 1.6.3.1	Membrane associated enzymes which hydrolyse NADPH to convert oxygen into superoxide. Cytosolic enzyme activity.	Cytosol (membrane associated)	NOX1 - Tissue enhanced: gastrointestinal tract, seminal vesicle.<br>NOX2 - Widely expressed; enriched in blood, lymphoid, lung.<br>NOX3 - Adrenal gland.<br>NOX4 - Widely expressed; enriched in kidney.<br>NOX5 - Tissue enhanced: spleen, testis, placenta.
**Dual Oxidases**	DUOX1<br>DUOX2	EC 1.6.3.1	NADP(H)/FAD(H_2) linked oxidase that generates H_2O_2 from O_2. Required for thyroid hormone synthesis.	Cytosol (membrane associated)	DUOX1 - Widely expressed; enriched in thyroid and lung.<br>DUOX2 - Widely expressed; enriched in thyroid, gall bladder, ductus deferens.
**Mitochondrial Electron Transport Chain**	ETC		Mitochondrial enzyme chain which converts energy from reducing intermediates into ATP. Can produce superoxide through forward or reverse electron transfer to oxygen at each of its complexes, though especially complexes I and III.	Mitochondria	Ubiquitous.
**Glycolate (hydroxyacid) Oxidase**	HAO1	EC 1.1.3.15	Oxidises glycolate to glyoxylate while producing H_2O_2. Peroxisomal.	Peroxisome	Liver
**Acyl-CoA Oxidase**	ACOX	EC 1.3.3.6	Catalyses peroxisomal β-oxidation and produces H_2O_2.	Peroxisome	ACOX1 – Ubiquitous.<br>ACOX2 – Ubiquitous; enriched in liver, kidney.<br>ACOX3 – Ubiquitous.<br>ACOXL - Widely expressed; enriched in lung, male reproductive system; placenta.
**Polyamine Oxidase**	PAO	EC 1.5.3.13	Oxidises compounds containing two positively charged amine groups, producing H_2O_2.	Peroxisome	Widely expressed; enriched in testis.
**Sarcosine Oxidase**	PSO	EC 1.5.3.1,<br>EC 1.5.3.7	Demethylates sarcosine to glycine, producing H_2O_2.	Peroxisome	Widely expressed; enriched in liver, kidney.
**Diamine Oxidases**	DAO<br>RAO	EC 1.4.3.22	Oxidises histamine, producing H_2O_2.	AOC1 – Secreted<br>AOC2 - Cytosol	DAO - Widely expressed; enriched in gastrointestinal tract, placenta.<br>RAO - Widely expressed; enriched in retina.
**Xanthine Oxidase**	XO	EC 1.17.3.2	Multifunctional oxidative enzyme. May generate superoxide from the oxidation of an R-H group to R-OH, a	Nucleus	Widely expressed; enriched in gastrointestinal tract, liver.

Supplementary Table S1: An outline of the majority of ROS generation, propagation and termination enzyme expressed in human cells.

			carbonate radical from the oxidation of acetaldehyde, or hydrogen peroxide from the oxidation of xanthine or hypoxanthine.		
**Protein Disulphide Isomerase**	PDI (PDIA1-19)	EC 5.3.4.1	Oxidises unfolded proteins in the endoplasmic reticulum to generate disulphide bonds. Some PDIs require thioredoxin as a cofactor.	Endoplasmic Reticulum	PDI (P4HB) – Ubiquitous; enriched in liver, pancreas.<br>PDIA2 – Pancreas.<br>PDIA3 – Ubiquitous<br>PDIA4 - Ubiquitous; enriched in liver<br>PDIA5 - Ubiquitous; enriched in liver<br>PDIA6 – Ubiquitous<br>PDIA7 (PDILT) – Testis, stomach.<br>PDIA8 (ERP27) – Enriched in pancreas<br>PDIA9 (ERP29) – Ubiquitous<br>PDIA10 (ERP44) – Ubiquitous<br>PDIA11 (TMX1) – Ubiquitous<br>PDIA12 (TMX2) – Ubiquitous<br>PDIA13 (TMX3) – Ubiquitous<br>PDIA14 (TMX4) – Ubiquitous<br>PDIA15 (TXNDC5) – Ubiquitous<br>PDIA16 (TXNDC12) – Ubiquitous<br>PDIA17 (AGR2) – Widely expressed; enriched in gallbladder, seminal vesicle, ductus deferens, gastrointestinal tract.<br>PDIA18 (AGR3) – Widely expressed; enriched in fallopian tube, gastrointestinal tract.<br>PDIA19 (DNAJC10) - Ubiquitous; enriched in epididymis.
**Endoplasmic Reticulum Oxidoreductins**	ERO1α<br>ERO1β	EC 1.8.4.-	Regenerates oxidised PDI in the endoplasmic reticulum and produces hydrogen peroxide.	Endoplasmic Reticulum	ERO1α - Ubiquitous; enriched in oesophagus<br>ERO1β - Ubiquitous; enriched in pancreas
**Monoamine Oxidase A&B**	MAOA<br>MAOB	EC 1.4.3.4	Oxidises catecholamines, producing H_2O_2.	Mitochondria	MAOA – Ubiquitous<br>MAOB – Widely expressed; enhanced in liver
**ROS Transmuting Enzymes**					
**Superoxide Dismutase 1**	SOD1	EC 1.15.1.1	Dimeric enzyme containing copper and zinc as a cofactor, which transmutes superoxide into hydrogen peroxide.	Cytosol	Ubiquitous; enriched in liver
**Superoxide Dismutase 2**	SOD2	EC 1.15.1.1	Tetrameric enzyme containing manganese as a cofactor, which transmutes superoxide into hydrogen peroxide.	Mitochondria	Ubiquitous; enriched in skeletal muscle, granulocytes.

Supplementary Table S1: An outline of the majority of ROS generation, propagation and termination enzyme expressed in human cells.

**Superoxide Dismutase 3**	SOD3	EC 1.15.1.1	Extracellular tetrameric enzyme containing copper and zinc as a cofactor, which transmutes superoxide into hydrogen peroxide.	Secreted	Ubiquitous.
			**ROS Dismuting Enzymes**		
**Catalase**	Cat	EC 1.11.1.21	Peroxisomal enzyme converting H_2O_2 to water and O_2	Peroxisome	Ubiquitous; enriched in liver, granulocytes.
**Glutathione Peroxidase**	GPx1-8	EC 1.11.1.9	Enzyme using reduced glutathione as an electron donor to convert hydrogen peroxide to water	Cytosol (GPx1 also reported in mitochondria)	GPx1 – Ubiquitous.<br>GPx2 – Widely expressed; enriched in liver, gallbladder.<br>GPx3 – Widely expressed; enriched in kidney, thyroid.<br>GPx4 – Ubiquitous.<br>GPx5 – Epididymis, testis.<br>GPx6 – Epididymis.<br>GPx7 – Ubiquitous.<br>GPx8 – Ubiquitous.
**Peroxiredoxin**	Prx I-VI	EC 1.11.1.5	Dimerises to convert H_2O_2 to water. Intracellular organellar localisation varies among isoforms. Also depending on isoform, utilises thioredoxin, glutathione or ascorbic acid for recycling of activity.	Prx-I – cytosol (some nucleus)<br>Prx-II – cytosol<br>Prx-III – mitochondria<br>Prx-IV – Endoplasmic Reticulum, cytosol<br>Prx-V – Mitochondria<br>Prx-VI – cytosol, plasma membrane	Prx-I – Ubiquitous.<br>Prx-II – Ubiquitous.<br>Prx-III – Ubiquitous.<br>Prx-IV – Widely expressed, enriched in liver and pancreas.<br>Prx-V – Ubiquitous.<br>Prx-VI – Ubiquitous.
**Ferritin**	FTH1<br>FTL<br>FTMT	EC 1.16.3.1	A 24 subunit protein of heavy (FTH1) and light (FTL) chains. Major intracellular iron storage protein. Variation in ferritin subunit composition is tissue/cell dependent and affects activity. Has iron dependent ferroxidase activity to convert H_2O_2 to H_2O, and limits hydroxyl radical production from the Fenton reaction.<br>Mitochondrial ferritin with iron handling and ferroxidase activity,	FTH1/FTL – cytosol, microtubule associated<br>FTMT - mitochondria	FTH1 – Ubiquitous.<br>FTL – Ubiquitous; enriched in blood.<br>FTMT – Widely expressed; enriched in testis.
			**Oxidated Protein Handling Enzymes**		

Supplementary Table S1: An outline of the majority of ROS generation, propagation and termination enzyme expressed in human cells.

**Thioredoxin**	Trx1<br>Mt-Trx	EC 1.8.1.8	Reduces certain oxidised proteins via formation of an intramolecular disulphide bond.	Trx1 - cytosol (some nucleus)<br>Mt-Trx - mitochondria	Trx1 – Ubiquitous.<br>Mt-Trx – Ubiquitous.
**Glutathione Reductase**	GSR	EC 1.8.1.7	Converts oxidised glutathione to reduced glutathione	Cytosol	GSR – Ubiquitous.
**Sulfiredoxin**	Srxn1	EC 1.8.98.2	Reduces H_2O_2 oxidised cysteine residues on target proteins.	Cytosol	SRXN1 – Ubiquitous.
**Glutathione-S-Transferase**	(M)GSTs	EC 2.5.1.18	Large family of enzymes that conjugate reduced glutathione to oxidised molecules, including peroxidised lipids, for detoxification.	GSTA1 – cytosol<br>GSTA2 – cytosol, membrane associated<br>GSTA3 – cytosol<br>GSTA4 – cytosol, membrane associated<br>GSTA5 – cytosol<br>GSTK1 – peroxisome<br>GSTM1 – cytosol<br>GSTM2 – cytosol<br>GSTM3 – cytosol<br>GSTM4 – cytosol, membrane associated<br>GSMT5 – cytosol, membrane associated<br>GSTO1 – cytosol<br>GSTO2 – cytosol<br>GSTP1 – cytosol, mitochondria<br>GSTT1 – cytosol<br>GSTT2 – cytosol<br>GSTT4 – cytosol<br>GSTZ1 – cytosol<br>MGST1 – mitochondria<br>MGST2 – microsomal<br>MGST3 – endoplasmic reticulum, microsomal	GSTA1 – Widely expressed, enriched in adrenals, intestine, liver, kidney.<br>GSTA2 – Widely expressed, enriched in kidney, liver, pancreas.<br>GSTA3 – Tissue enhanced: adrenal, fallopian tube, placenta.<br>GSTA4 – Widely expressed; enriched in adrenal.<br>GSTA5 – Testis.<br>GSTK1 – Ubiquitous.<br>GSTM1 – Widely expressed, enriched in liver, seminal vesicle.<br>GSTM2 – Ubiquitous.<br>GSTM3 – Widely expressed, enriched in testis, seminal vesicle.<br>GSTM4 – Widely expressed; enriched gastrointestinal tract, granulocytes.<br>GSMT5 – Widely expressed, enriched in breast, ovary.<br>GSTO1 – Widely expressed, enriched in liver.<br>GSTO2 – Ubiquitous.<br>GSTP1 – Ubiquitous.<br>GSTT1 – Ubiquitous.<br>GSTT2 – Widely expressed, enriched in breast.<br>GSTT4 – Unknown<br>GSTZ1 – Widely expressed, enriched in liver.<br>MGST1 – Widely expressed, enriched in liver, adipose.<br>MGST2 – Ubiquitous.<br>MGST3 – Widely expressed, enriched in heart.
**Epoxide Hydrolases**	mEH<br>cEH<br>EH3-4	EC 3.3.2.10	Hydrolyses oxidised compounds which have formed epoxide groups.	mEH – endoplasmic reticulum<br>cEH – cytosol, peroxisome<br>EH3 – microsomal<br>EH4 – microsomal	mEH – Widely expressed, enriched in liver, adrenal.<br>cEH – Widely expressed, enriched in liver, intestine.<br>EH3 – Tissue enhanced: oesophagus, tongue, skin, lymphoid tissue.<br>EH4 – Tissue enhanced: brain, T-cells.
